# Supplementary material for: Lung and Gut Microbiota Changes Associated with Pseudomonas aeruginosa Infection in Mouse Models of Cystic Fibrosis
Source: Int J Mol Sci. 2021 Nov 10;22(22):12169. doi: 10.3390/ijms222212169 (PMC8625166; doi:10.3390/ijms222212169)
Supplement: Supplementary file 1 [file ijms-22-12169-s001.zip › Bacci et al. Supplementary Materials_IJMS 2021_special_issue_revised.pdf]

International Journal of *Molecular Sciences*

MDPI

Supplementary Materials

Lung and Gut Microbiota Changes Associated with *Pseudomonas Aeruginosa* Infection in Mouse Models of Cystic Fibrosis

Giovanni Bacci [1], Alice Rossi [2], Federica Armanini [3], Lisa Cangioli [1], Ida De Fino [2], Nicola Segata [3], Alessio Mengoni [1], Alessandra Bragonzi [2] and Annamaria Bevivino [4,*]

[1] Department of Biology, University of Florence, Sesto Fiorentino, 50019 Florence, Italy; giovanni.bacci@unifi.it (G.B.); lisa.cangioli@unifi.it (L.C.); alessio.mengoni@unifi.it (A.M.)
[2] Infections and Cystic Fibrosis Unit, Division of Immunology, Transplantation and Infectious Diseases, IRCCS San Raffaele Scientific Institute, 20132 Milan, Italy; rossi1.alice@hsr.it (A.R.); defino.ida@hsr.it (I.D.F.); bragonzi.alessandra@hsr.it (A.Br.)
[3] Department CIBIO, University of Trento, 38122 Trento, Italy; federica.armanini@unitn.it (F.A.); nicola.segata@unitn.it (N.S.)
[4] Department for Sustainability, Italian National Agency for New Technologies, Energy and Sustainable Economic Development, ENEA Casaccia Research Center, 00123 Rome, Italy; annamaria.bevivino@enea.it (A.B.)
* Correspondence: annamaria.bevivino@enea.it; Tel.: +39-0630-483-868

Keywords: cystic fibrosis; lung; gut; microbiome; gut-lung axis; animal models; CFTR mice; Pseudomonas aeruginosa

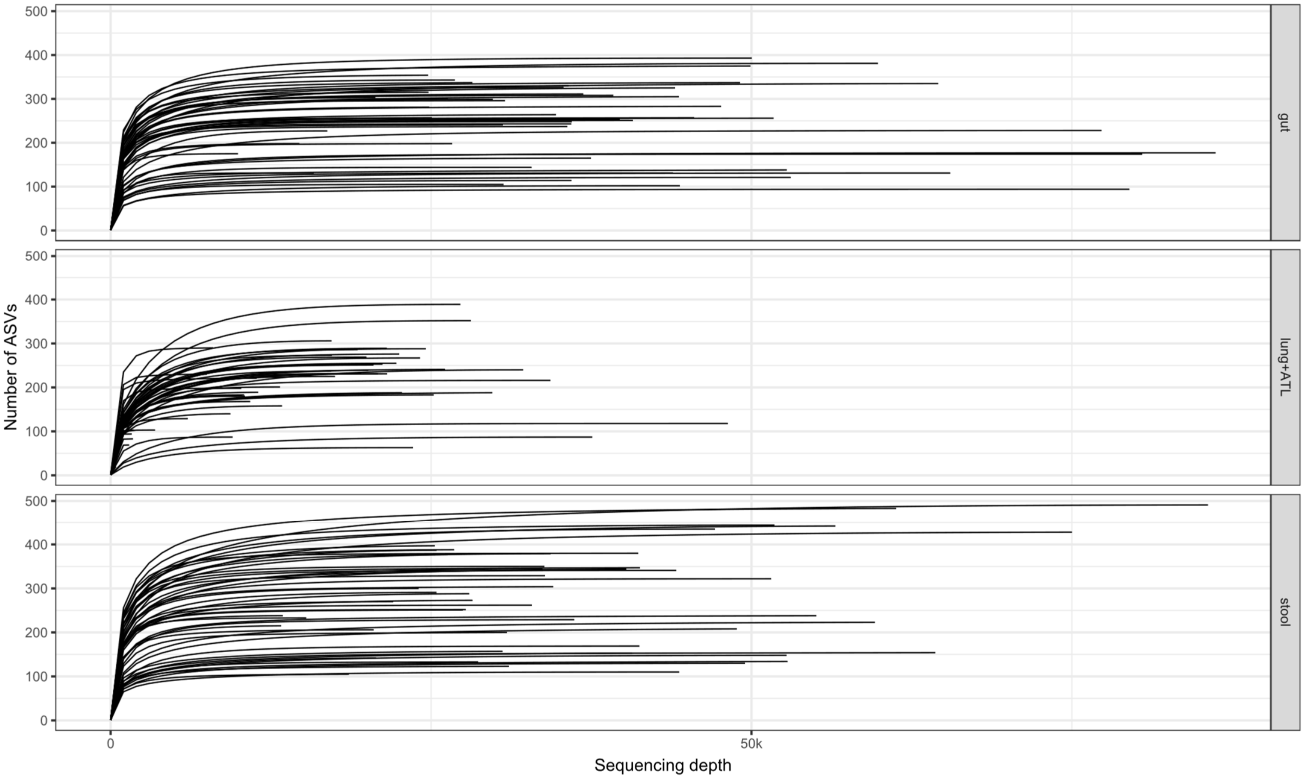

Figure S1: Rarefaction curves of ASVs

Table S1: Reads statistics and Good's coverage of samples
Table S2: Taxonomic assignment of the Amplicon Sequence Variants (ASVs)
Table S3: Differential abundance of ASVs from Deseq2 analysis
